# Revealing the Prevalence of *Toxoplasma* in Sierra Morena’s Wild Boar: An ELISA-Based Study Using Meat Juice

**DOI:** 10.3390/pathogens13040281

**Published:** 2024-03-26

**Authors:** José María Castillo-Castillo, Pablo José Rufino-Moya, Álvaro Martínez-Moreno, Ángela Salvador Castaño, Francisco Javier Martínez-Moreno, Rafael Zafra Leva

**Affiliations:** Departamento de Sanidad Animal (Parasitología), Facultad de Veterinaria, Universidad de Córdoba, Edificio de Sanidad Animal, Campus de Rabanales, Ctra. Madrid-Cádiz km 396, 14014 Córdoba, Spain; j.m.castillo@icloud.com (J.M.C.-C.); pablo.rufino.moya@gmail.com (P.J.R.-M.); amm@uco.es (Á.M.-M.); angelasalcas@gmail.com (Á.S.C.); rafael.zafra@uco.es (R.Z.L.)

**Keywords:** wild boar, *Toxoplasma gondii*, meat juice, ELISA

## Abstract

This research work focused on the prevalence of *Toxoplasma gondii* in wild boar from the Sierra Morena region. We conducted an ELISA analysis using meat juice samples. A total of 892 samples from six hunting seasons (2013–2019) were collected from the provinces that constitute the Sierra Morena Mountain range. These samples were analyzed using the Pigtype^®^ ELISA kit, specifically developed for detecting *T. gondii* in meat juice. The overall prevalence of *T. gondii* in Sierra Morena was 23.2%. The highest prevalences were observed in Córdoba (31.6%) and Jaén (25.9%). These provinces exhibit the highest density of wild boar as well as the greatest presence of the Iberian lynx (*Lynx pardinus*). Further in-depth studies are necessary, but it appears that the presence of wild felids and scavenger behavior may be associated with this observation.

## 1. Introduction

Toxoplasmosis, caused by the protozoan *Toxoplasma gondii*, is a parasitic zoonosis that is widely spread throughout the world. This parasite has a complex life cycle, in which domestic and wild felines act as definitive hosts. This cycle can involve virtually all warm-blooded vertebrates, including mammals and birds, as intermediate hosts [1]. In our region, cats and lynxes serve as definitive hosts in the complex life cycle of *T. gondii*, excreting millions of oocysts in their feces. These oocysts can infect intermediate hosts through the contamination of soil, water, or food [2,3,4,5]. However, *T. gondii* can also circulate in the environment without the involvement of definitive hosts (via tissue cysts in the tissues of intermediate hosts), as well as without the involvement of intermediate hosts (via environmentally contaminated oocysts) [6].

Toxoplasmosis is one of the primary foodborne zoonoses and can even be considered the second most important foodborne parasite in Europe [7,8]. Nearly 30% of the world’s human population has had contact with the parasite, as evidenced by the presence of anti-*T. gondii* antibodies [9,10,11,12]. Some studies suggest that between 30% and 50% of the global human population is infected with *T. gondii* [13]. Humans can become infected via three routes: by ingesting sporulated oocysts that contaminate the environment, by consuming raw or undercooked meat containing parasite tissue cysts, and by intrauterine infection [14]. 

Demand for game meat is on the rise in Europe, as it is perceived to be a sustainable, healthy, and ecologically friendly product. However, this increase in demand raises the potential for an increase in the transmission of food-borne pathogens associated with wildlife, including *T. gondii*, if the meat is consumed raw or undercooked [9,15,16,17,18,19,20,21]. Wild boar, in contact with *T. gondii* from their local environment, could serve as indicators for understanding the geographical variations of the parasite [22]. The species plays a significant role as one of the most important scavengers in the Mediterranean ecosystem [23,24], thereby exposing themselves to an increased risk of infection due to this trophic behavior. Conversely, the rearing conditions of Iberian pigs, characterized by long periods of pasture feeding and limited biosecurity measures, could promote greater contact between *T. gondii* and these pigs [25]. This escalates the risk of infection through pork, which is considered the greatest risk of infection in humans [26].

Spain ranks third in Europe in the number of wild boar (*Sus scrofa*) hunted per year [27]. More than 370,000 wild boar are hunted annually in this country, generating an economic impact of 22 million euros [28]. 

The prevalence of *Toxoplasma* infection in wild boar is influenced by factors such as the age and sex of the animals as well as the season and weather conditions [29,30]. Prevalence varies in different European countries: 40% (range 32–59%) in the Czech Republic [31]; 48% in Poland [32]; 29% (range 5–45%) in Sweden [33]; 16.8% in France [34]; 62% in Slovenia [35]; 35% in Switzerland [36]; and 15.5% in Italy [37]. In Spain, prevalence also varies between different areas: 14.1% in the Valencian Community [30]; 39% in Doñana National Park [29]; 19.4% in Andalusia [38]; 23.88% in the Extremadura Region and 20.8% in the Sierras de Cazorla, Segura, and Las Villas Natural Park [39]; and 51.16% in Sierra Morena [40]. Only three studies focus on the Sierra Morena area, as is the case of the present study. However, there are limited data about the prevalence of *T. gondii* infection in wild boar from Sierra Morena, a mountain range in southern Spain, which is an important area for hunting activities and a natural habitat for many wild animals living with livestock.

## 2. Materials and Methods

### 2.1. Study Area 

**Figure 1:**
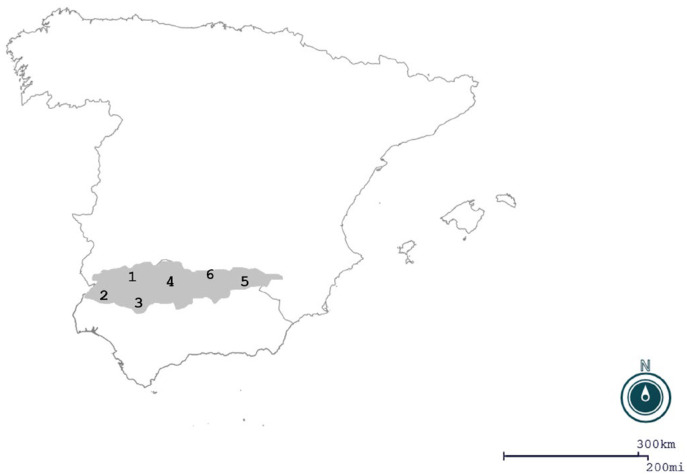
Study area. The area in grey corresponds to the Sierra Morena Mountain. Numbers indicate the provinces: Badajoz (1); Huelva (2); Seville (3); Córdoba (4); Jaén (5); and Ciudad Real (6). Map created with Epi Info^®^ v. 7.2.6.0.

As mentioned previously, the hunting area encompasses the Sierra Morena Mountain range, located in the southern part of Spain (Figure 1).

### 2.2. Samples and Sample Collection

A total of 892 yearlings and adult wild boar were sampled from 2013 to 2019, spanning six different hunting seasons that started at the beginning of October and ended at the end of February. These samples came from six provinces (Badajoz, Huelva, Seville, Córdoba, Jaén, and Ciudad Real) that belong to the Sierra Morena area.

Samples from wild boar were obtained from animals that had been legally hunted by authorized hunters possessing the appropriate permits and licenses, and with landowner consent. Sampling was conducted during the hunting season, in compliance with Spanish and EU legislation (UE 2015/1375). It should be noted that no animals were specifically hunted for the purposes of this study, and, therefore, ethical approval by an Institutional Animal Care and Use Committee was not deemed necessary. 

Samples were randomly collected at a game meat processing plant, adhering to a consistent sampling procedure. This procedure entailed excising a piece weighing approximately 30 g from the left front leg flexor muscles. The sample was then packaged, labelled, and transported to a laboratory for further analysis. Meat juice samples were derived by freezing approximately 10 g of the original sample (the 30 g portion consisting of blood and fat-free muscle meat). The meat juice was procured by compressing the frozen muscle and it was then deposited into an Eppendorf tube. Each sample was labelled to ensure traceability and subsequently stored at −20 °C until analysis.

### 2.3. Laboratory Analysis

Analysis of the meat juice was conducted using the Pigtype^®^ *Toxoplasma* Ab kit (Qiagen, Leipzig, Germany), in accordance with the manufacturer’s instructions. The Pigtype^®^ *Toxoplasma* Ab kit has showed a high specificity (100%) and sensitivity (89.3%). It is an ELISA assay designed for the detection of *T. gondii* antibodies in serum, plasma, and meat juice samples, specifically from pigs and wild boar, among other species.

In brief, a working solution of Washing Fluid was prepared by diluting the initial solution tenfold with demineralized water. Meat juice samples were diluted at a 1:10 ratio in Sample Diluent. Both positive and negative controls (provided by the manufacturer in the kit) were added in duplicate to each plate. Each well was filled with 100 μL of either sample or control. The plate was incubated for 60 min at room temperature (15–25 °C) after which each well was washed three times with 300 μL of the prepared Wash Buffer. A volume of 100 μL of ready-to-use Conjugate was added to each well, followed by a 30-min incubation at room temperature. The wells were subsequently washed three times. Thereafter, 100 μL of the substrate (TMB) was added to each well, and the plate was incubated for 10 min at room temperature in the dark. The reaction was halted by adding 100 μL of Stop Solution to each well. The optical density (OD) was measured using a spectrophotometer Multiskan^®^ FC (Thermo Fisher Scientific^®^, Paisley, UK) at 450 nm. 

### 2.4. Statistical Analysis

Descriptive parameters were obtained using QPweb (Quantitative Parasitology on the Web) version 1.0.15 [41] and the Winepi website (www.winepi.net/sp/index.htm, accessed on 10 January 2024). QPweb was utilized to calculate prevalence with a 95% confidence interval (95% CI) using Sterne’s exact method. Subsequently, the prevalence was adjusted to the specificity and sensibility values of the Pigtype^®^ Toxplasma Ab kit as provided by the Winepi website.

Statistical comparisons were performed using the Winepi website (www.winepi.net/sp/index.htm, accessed on 10 January 2024) and Jamovi software v. 2.3 (The Jamovi Project, 2023). For general, inter-province, and intra-province comparisons, a Chi-square test was employed, again utilizing the Winepi website. 

Additionally, a correlation study was conducted between the number of samples and the number of positive results. Depending on the distribution, the Pearson/Spearman tests were used, both implemented in Jamovi Software v. 2.3.

In all instances, a *p*-value of less than 0.05 was considered statistically significant.

## 3. Results

### 3.1. T. gondii Prevalence

Results regarding the overall prevalence in Sierra Morena, as well as for each province studied, are showed in Table 1. As commented below, the prevalence was calculated considering the specificity and sensitivity values of the Pigtype^®^ *Toxoplasma* Ab Kit [42].

As can be observed from the table below, there was a notable heterogeneity in both the number of samples collected and the prevalence across provinces. These values ranged from a low of 12.9% in Seville to a high of 31.6% in Córdoba. A correlation study was performed to ensure the absence of bias related to the number of samples and the number of positive results (r = 0.8, *p* = 0.33), validating the prevalence data. For ease of data analysis, the samples were categorized into two groups: Western Sierra Morena (comprising Badajoz, Huelva, and Seville) and Eastern Sierra Morena (comprising Córdoba, Jaén, and Ciudad Real). The results of this categorization are presented in Table 2.

The statistical analysis conducted to compare the prevalence between Sierra Morena (Total) and the categorization into Western and Eastern regions revealed no significant differences. This was determined by applying the Chi-square test to compare proportions, yielding *p*-values of 0.25 and 0.37 for the Western and Eastern regions, respectively.

When the prevalence of the different provinces was compared to the prevalence observed in Sierra Morena (Total), only Córdoba showed a significantly (*p* = 0.03) higher prevalence in comparison to the whole region studied.

### 3.2. Inter-Province Comparisons

An inter-province comparison was performed to determine whether, in addition to Córdoba, there were other provinces with a significantly higher prevalence. According to the Chi-square test, three provinces exhibited significant differences in prevalence: Córdoba, Jaén, and Ciudad Real. Specifically, Córdoba demonstrated a significantly higher prevalence compared to Huelva (*p* = 0.01), Seville (*p* = 0.05), and Ciudad Real (*p* = 0.003). On the other hand, Jaén showed a significant increase only when compared to Ciudad Real (*p* = 0.003). However, the remaining comparisons between provinces were not statistically significant.

### 3.3. Intra-Province Comparisons

Subsequently, an intra-province comparison was conducted to determine if there was a hunting season with a significantly higher prevalence within the same province. For this purpose, the Chi-square test was applied to compare the prevalence in the different hunting seasons from 2013 to 2019 with the average prevalence of the province (Table 1). The results derived from this analysis are presented in Table 3. Significant increases in prevalence were found in three provinces: (1) Huelva (Western Sierra Morena) showed a significant increase (*p* = 0.02) in 2017–2018 (34.16%); (2) Córdoba (Eastern Sierra Morena) also showed a significant increase beginning in 2015–2016 (*p* = 0.02), with the highest prevalence in 2016–2017 (55.99%); similar results were observed for Badajoz, with a significant increase (*p* = 0.01) beginning in 2016–2017, and the highest prevalence in 2017–2018 (44.79%); and, finally, (3) Jaén (Eastern Sierra Morena) showed the highest seroprevalence (*p* = 0.01) in 2013–2014 (46.10%). 

## 4. Discussion

For many zoonotic parasitic species, studying their prevalence in wild animals serves as a significant indicator of the extent of parasitosis and the risk of transmission to humans.

In the case of game animals, where access is typically limited to the deceased animal, obtaining blood samples can be challenging. Therefore, the analysis of meat juice samples is employed. These samples can be collected during the animal’s inspection, from the carcass, or even only from access to parts of the animal [43].

In addition to the convenience of sample collection, it has been demonstrated that there is a strong correlation between the antibodies present in blood and those detected in meat juice. This correlation ensures the reliability of the test [44] and is considered suitable for use in various animal species and parasites [14,45]. In the case of *Toxoplasma*, the use of meat juice has demonstrated high values of sensitivity and specificity [42]; it has thus been successfully used as an appropriate matrix for monitoring antibodies against *T. gondii* [14,46,47,48]. In fact, recently, this technique has been used for the diagnosis of *Toxoplasma* in wild boar in the Czech Republic [31], in France [34], in Poland [32], and in Switzerland [36]. 

In agreement with previous authors [7,21,32,36,49], *T. gondii* infection in wild boar may represent a potential source of infection for humans.

Given the importance of wild boar in Spain, particularly as game meat, it becomes crucial to update our understanding of the parasitosis in this species. In Spain, wild boar are not only found in natural or wild areas but also in peri-urban regions, where these boar encounter extensive Iberian pig farms [25]. This proximity between wild boar and domestic pigs necessitates vigilance. 

The ecological niche where wild boar thrive is conducive to infection through the ingestion of cysts and oocysts. This niche is shared with wild felids and other intermediate hosts through carnivorism.

Dedicated research specifically focused on *T. gondii* infection in the wild boar population is limited in Spain [29,40,50], and there are few data on toxoplasmosis in the wild boar. However, these data are primarily embedded within studies that investigate other pathogens in the wild boar [51,52]. These studies were conducted in different environments from the one addressed in our current work.

To the author’s knowledge, this is the first study to provide data on *Toxoplasma* infection in an area of special ecological and hunting interest, extending from west to east in southern Spain.

The overall prevalence obtained in this study is 23.2%. This result is lower than that observed at national level (36% and 38.5%) [40,51] and in Catalonia (43.5%) [52]. It should be noted that these studies use serum from heart blood and were analyzed by MAT. The comparison between our study and these studies reveals the variation observed in Spain according to the sampling area, suggesting a dependence on the geographical region and the type of hunting area. Perhaps this could be the reason why in our study a lower value of prevalence was found. If we pay attention to studies performed only in southern Spain, our result is also lower than that describe previously, a prevalence of 39% [29], though higher than other previous reports (18.6% and 20.8%, respectively) [38,39]. Again, it should be noted that these studies use serum samples obtained from the thoracic cavity or endocranial venous sinus and were analyzed by MAT. Results similar to our study were observed in Cáceres, where a prevalence of 23.8% was found using serum samples analyzed by ELISA [50]. The technique employed in their study was closely related to the one used in our research.

At a European level, the prevalence of *Toxoplasma* in wild boar varies depending on the region and the technique, ranging from 5.2% in Greece [53] to 62% in Slovenia [35]. The lack of a perfect technique (with 100% specificity and 100% sensitivity) for the detection of *T. gondii* is well-known [54]. However, there are differences and variations in both the technique and the type of sample and method employed [55]. In our study, we chose to use meat juice as the sample because the origin of the animal prevented us from using blood or plasma; meat juice was the best available option. As we explained in the “material and methods” section, the values of specificity and sensitivity are sufficiently high to ensure the accuracy of the analysis.

In this context, blood samples from hunted animals were analyzed in several countries using various techniques. In Sweden, ELISA analysis revealed a prevalence of 50% (29–65%) [33,56]. In Poland, MAT analysis of abdominal cavity blood showed a 37% prevalence [49], with regional variations of 11.6–50%. In Germany, ELISA analysis of abdominal cavity samples showed a mean prevalence of 24.4% [57]. In Denmark, ELISA analysis of blood from deceased animals showed a mean prevalence of 27.7% (19.7–37.1%) across five regions [7]. Slovenia reported the highest prevalence (62%) in serum from deceased animals [35]. Except for the case of Germany, these values are higher than those observed in our study, potentially due to differing climatic conditions in southern Spain compared to these northern countries. Additionally, some studies reported regional variations in prevalence [7,33,56], similar to the heterogeneity we found in the prevalence across the different provinces investigated, with higher values observed in Córdoba, Badajoz, and Jaén. 

Turning our attention to meat juice samples, these have been analyzed by ELISA and reported a prevalence of 48% in Poland [32], 40% in the Czech Republic [31], and 35% in Switzerland [36]. Despite using the same type of sample and technique, these results are higher than those observed in our study. This supports the hypothesis below, that the different prevalences observed could be related to the climatic features of these northern countries. In fact, it should be noted that some of these studies found regional differences in prevalence, ranging from 32–59% in the Czech Republic [31] and 29–37% in Switzerland [36]. In our study, we also found differences in prevalence in some provinces of Sierra Morena, with higher prevalences of *Toxoplasma* in Córdoba (31.6%), Badajoz (24.8%), and Jaén (25.9%). 

As previously mentioned, our study found no significant differences between Eastern and Western Sierra Morena. However, we observed some variation in relation to the province, with prevalences ranging from 12.9% (Seville) to 31.6% (Córdoba). Therefore, it is believed that environmental, geographical, and climatic conditions can significantly influence the occurrence of toxoplasmosis [58]. Additionally, there are other factors that could contribute to these differences:

1. The area hosts definitive species such as the lynx, along with both domestic and wild felids. Recent studies demonstrate the involvement of the Eurasian lynx in the *Toxoplasma* cycle, acting as both an intermediate and definitive host. This is evidenced by the detection of cysts and the elimination of oocysts [4]. This felid is common in Sierra Morena, where the two largest populations of the Iberian lynx (*Lynx pardinus*) are found; specifically, in two of the provinces under study (Córdoba and Jaén) [59]. These areas coincide with the highest prevalence of *Toxoplasma* in the study. Therefore, the presence of Iberian Lynx has been positively correlated with *Toxoplasma* infection in other wild species, such as red deer [29], and is also associated with proximity to other felids, whether wild or domestic [25]. This presence may explain the high prevalence of *Toxoplasma* in other wild ungulates in southern Spain, as observed in various studies [38,39]. In the case of Badajoz, the cause could be in the presence of wildcat populations. These felids have been identified in central Spain, which may also contribute to the epidemiology of *Toxoplasma*, as their contact with the parasite has been demonstrated by PCR, and their ability to eliminate oocysts, albeit in small numbers, has also been identified [60].
2. The ingestion of cysts present in intermediate hosts is a key factor. Studies performed in other wild felids [4] have reported low shedding of oocysts in these animals. This suggests a limited presence of oocysts in the environment and indicates that the main source of infection is the ingestion of cysts present in rodents, carcasses, or infected visceral organs of domestic animals [31,61,62].

The survival of oocysts is influenced by climatic and environmental conditions, particularly temperature and rainfall [29,30,31].

Regarding to this, our study found no statistical differences in temperature and rainfall between provinces in Sierra Morena.

3. The density of animals in a specific area is a critical factor. It has been observed that the prevalence of Toxoplasma increases with the density of wild boar [29,31,40]. These factors cause the prevalence to fluctuate seasonally, with significant differences depending on these conditions. This phenomenon has been demonstrated in France [34] and Denmark [7]. In our study, the highest densities of wild boar relative to the number of animals hunted during the years under consideration were found in Córdoba and Jaén (https://www.juntadeandalucia.es/medioambiente/portal/acceso-rediam/estadisticas/estadisticas-oficiales/caza-y-actividades-cinegeticas-andalucia, accessed on 1 December 2023). This finding supports the hypothesis regarding the correlation between animal density and prevalence, and it reinforces the hypothesis that addresses the causes behind the higher prevalences found in these provinces.

## 5. Conclusions

This study investigates the presence of *T. gondii* in wild boar populations within the Sierra Morena area. To the best of the author’s knowledge, this is one of the few studies that analyzes this specific aspect. The research encompasses a substantial sample size of 892 wild boar, collected over six hunting seasons (2013–2019). The results reveal a prevalence rate of 23.2% in the Sierra Morena region. Interestingly, this value closely aligns with findings from previous studies conducted on Iberian pigs in southern Spain. This correlation is logical, given the integration between wild boar and Iberian pigs. Notably, two provinces, Córdoba (31.6%) and Jaén (26.9%), exhibit the highest prevalence rates. These figures coincide with the regions where wild boar hunting is more intense and where the Iberian Lynx (*Lynx pardinus*) population census is also elevated. Further in-depth investigations are warranted, but it appears that the density of wild boar populations (and their scavenging behavior) and the presence of wild felids may contribute to these elevated prevalence rates.

## Figures and Tables

**Table 1:** Total number of samples analyzed, number of positives, and prevalence (expressed as percentage) for each province within the study area (Sierra Morena). The 95% confidence interval is shown in brackets.

Province	Samples (*n*)	Positives	Prevalence (CI 95%)
Badajoz	54	12	24.8% (14.3–39.5)
Huelva	299	56	20.9% (16.4–26.2)
Seville	26	3	12.9% (3.5–34.1)
Córdoba	138	39	31.6% (23.7–40.8) ^†^
Jaén	229	53	25.9% (20.2–32.5)
Ciudad Real	146	22	16.8% (11.1–24.4)
**TOTAL**	**892**	**185**	23.2% (20.2–26.3) *

* Average value; ^†^ Significant difference with the total prevalence observed in the area.

**Table 2:** Prevalence data for the entire study area (Sierra Morena), as well as for the province within Western and Eastern Sierra Morena.

Location	*n*	Positives	Prevalence	IC 95%	
				Lower	Upper
Sierra Morena (Total)	892	185	23.2	20.2	26.3
Sierra Morena (Western) ^1^	375	68	20.3	16.2	25.1
Sierra Morena (Eastern) ^2^	517	117	25.3	21.4	29.6

^1^ Badajoz, Huelva, and Seville. ^2^ Córdoba, Jaén, and Ciudad Real.

**Table 3:** Results derived from intra-province comparisons. Data of prevalence is presented as a percentage. In brackets: (number of samples analyzed/number positive results).

Season	Badajoz	Huelva	Seville	Córdoba	Jaén	Ciudad Real
2013–2014	0% (6/0)	15.99% (91/13)	0% (7/0)	22.39% (20/4)	46.10% * (34/14)	11.19% (20/2)
2014–2015	0% (6/0)	25.28% (62/14)	0% (3/0)	23.75% (33/7)	20.56% (49/9)	21.43% (47/9)
2015–2016	6.58% (17/1)	11.19% (20/2)	74.65% *,† (3/2)	51.68% * (13/6)	16.58% (27/4)	6.99% (16/1)
2016–2017	32.93% * (17/5)	0% (25/0)	0% (0/0)	55.99% * (22/11)	23.16% (29/6)	19.19% (35/6)
2017–2018	44.79% * (5/2)	34.16% * (59/18)	0% (5/0)	22.39% (45/9)	20.35% (44/8)	21.32% (21/4)
2018–2019	37.32% (3/1)	23.99% (42/9)	13.99% (8/1)	44.79% (5/2)	29.20% (46/12)	0% (7/0)

* Significant increases within the province. † Unreliable due to the small sample size according to the statistical test.

## Data Availability

Dataset available on request from the authors.
